# Motivating Self‐Care and Enhancing Treatment Adherence in Elderly Type 2 Diabetes: An Extended Parallel Process Model Intervention

**DOI:** 10.1155/nrp/9114238

**Published:** 2025-12-17

**Authors:** Malihe Kabusi, Gholam Reza Mahmoodi-Shan, Abdurrhman Charkazi, Mahin Tatari

**Affiliations:** ^1^ Nursing Department of Faculty of Nursing and Midwifery, Golestan University of Medical Sciences, Gorgan, Golestan, Iran, goums.ac.ir; ^2^ Nursing Research Center, Golestan University of Medical Sciences, Gorgan, Golestan, Iran, goums.ac.ir; ^3^ Department of Public Health, Faculty of Health, Golestan University of Medical Sciences, Gorgan, Golestan, Iran, goums.ac.ir; ^4^ Biostatistics Department, Golestan University of Medical Sciences, Gorgan, Golestan, Iran, goums.ac.ir; ^5^ Sapienza University of Rome, Rome, Lazio, Italy, uniroma1.it

## Abstract

**Background:**

This study aimed to examine the effectiveness of an intervention based on the Extended Parallel Process Model (EPPM) in improving treatment adherence and self‐care among elderly individuals with type 2 diabetes.

**Methods:**

This randomized clinical trial was conducted among 70 elderly individuals with type 2 diabetes attending the Deziani Diabetes Clinic in Gorgan, Iran, during 2022. Participants were aged 60 years or older and capable of participating in the study. They were randomly assigned to either an intervention or a control group. The intervention was delivered over 5 weeks, and a posttest was conducted 4 weeks after the final session.

**Results:**

The Mann–Whitney *U* test revealed a statistically significant difference in treatment adherence between the intervention and control groups (*U* = −5.78, *p* = 0.01). The intervention group showed higher treatment adherence (mean = 102.91 SD = 6.67) than the control group (mean = 88.51, SD = 10.63), with a confidence interval of 11.18, a *p*‐value of 0.001, and an effect size of *η* = 0.61 (*p* < 0.01), indicating that approximately 61% of the posttest variance was attributable to the intervention. There was a significant difference between total self‐care scores after the intervention (*t* = 6.66, *p* = 0.01, CI: 8.31, 15.4). These findings suggest that the EPPM‐based intervention effectively improved treatment adherence among elderly individuals with type 2 diabetes.

**Conclusion:**

The EPPM‐based educational intervention significantly improved treatment adherence and self‐care among elderly patients with type 2 diabetes. These results support the integration of the EPPM framework into diabetes education and management programs.

**What This Paper Adds**

• The Extended Parallel Process Model is an effective training program for improving treatment adherence in diabetic patients.
• Extended Parallel Process Model is also an effective training program for promoting self‐care in diabetic patients.
• Theory‐based educational interventions are essential for changing behavior.

## 1. Introduction

Old age is the final stage of human life. Developing countries currently accommodate approximately 60% of the world’s elderly population [1]. Iran’s elderly population has grown significantly, comprising about 10.7% of the total population according to the 2021 census [2]. Projections suggest a further increase to 24% by 2050, positioning Iran as a key country facing global aging challenges. This demographic shift presents considerable economic, social, and health burdens. Aging is associated with physiological deterioration and an increased susceptibility to acute and chronic diseases compared with younger populations [3].

Type 2 diabetes is a common chronic condition among the elderly, especially those aged 60 years and older. The incidence rate of type 2 diabetes is 9.8 per 1000 person‐years and is associated with lower educational levels [4], which significantly reduces the quality of life. Notably, nearly one‐fifth of individuals aged 65 years and older have type 2 diabetes, with incidence rates having increased sharply over the past decade [5]. Effective diabetes management relies on adherence to treatment regimens and a thorough understanding of the disease. Insufficient awareness and noncompliance with treatment are major factors contributing to inadequate diabetes care [6]. Adherence is essential for controlling diabetes and reducing the risk of complications [7]. It is defined as following medical advice and healthcare recommendations, including taking prescribed medications, adhering to dietary guidelines, and making necessary lifestyle modifications*,* and is a key determinant of successful diabetes management [8]. Poor adherence is associated with higher rates of hospitalization, treatment interruptions, increased healthcare costs, and more frequent doctor visits [9]. Conversely, good patient adherence helps prevent complications and reduces the economic burden of diabetes [10].

Self‐care is a crucial component of diabetes management and the prevention of complications, complementing treatment adherence [11]. Patient education plays a vital role in improving both treatment adherence and self‐care practices [12]. By focusing on knowledge‐driven behavioral changes, self‐care serves as a fundamental strategy that enhances patient health and reduces diabetes symptoms [13]. Self‐care refers to an individual’s proactive approach to maintaining health, preventing disease, and managing chronic conditions. It includes actions aimed at preventing both short‐ and long‐term complications [14]. Inadequate self‐care can lead to severe health outcomes; therefore, self‐care is a cornerstone of diabetes management [12]. Educational and behavioral training programs designed to promote treatment adherence often simultaneously strengthen patients’ self‐care capabilities [15].

Theory‐based educational interventions are pivotal for promoting behavior change [16]. The Extended Parallel Process Model (EPPM) is a prominent framework for developing effective health communication strategies and encouraging the prevention of risky behaviors. Proposed by Kim Witte in 1992, the EPPM is a theory of fear‐based motivation [17]. Fear‐inducing messages trigger evaluations of both the perceived threat and perceived efficacy. Individuals who perceive either low threat or low efficacy tend to ignore health messages because of inadequate motivation to change behavior [18]. Studies have consistently demonstrated the effectiveness of the EPPM in addressing various health issues. For example*,* Quick et al. found that EPPM‐based interventions significantly reduced the risk of noise‐induced hearing loss [19], while Abril et al. reported improved outcomes in smoking prevention [20]. Unlike other health education models, the EPPM integrates motivational and cognitive components by emphasizing perceived risk and efficacy, thereby fostering a more comprehensive understanding of health threats and protective behaviors [21].

Diabetes is a common chronic condition among the elderly, and education is crucial for its effective management and the prevention of related complications. Self‐care plays a significant role in maintaining independence and overall health in this population. Due to the limited research on the impact of educational interventions on self‐care among elderly individuals with type 2 diabetes in Golestan Province, northern Iran, this study aimed to evaluate the effect of an EPPM‐based intervention on treatment adherence and self‐care among elderly individuals with type 2 diabetes at the Diabetes Clinic in Gorgan, Iran.

## 2. Methods

**Figure 1:**
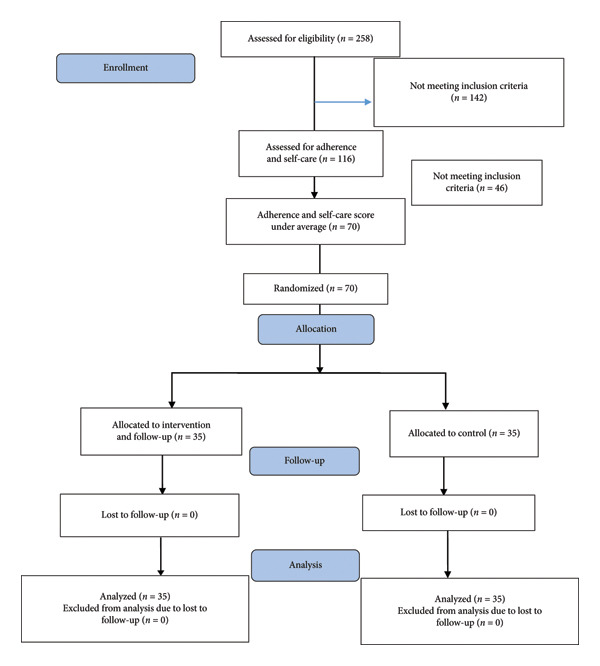
Flowchart of sample allocation in diabetic patients type 2 according to the CONSORT 2010 flow diagram.

This study utilized a randomized clinical trial with a pretest, posttest design and a single‐blind control group (statistician blinded) (Figure 1). The intervention group received five 45 min training sessions based on the EPPM. The control group received routine nursing care and education provided by the setting. The study was conducted between May and August 2022 at the Deziani Diabetes Clinic in Gorgan City, Iran. Participants were elderly individuals with type 2 diabetes aged 60 years or older, without psychiatric conditions, and capable of understanding educational materials. Exclusion criteria included prior participation in similar educational programs. Based on previous research [11, 22] and statistical power calculations, a sample size of 70 participants (35 per group) was determined to be adequate for detecting significant differences in treatment adherence and self‐care outcomes.

A total of 116 eligible elderly patients with type 2 diabetes were identified from the clinic’s patient list. Eligible participants completed self‐administered questionnaires assessing treatment adherence and self‐care. From those scoring below average on both measures, 70 individuals were randomly assigned to either the intervention or control group. Randomization was performed using a random number table from a standard statistics text. One of the research team members conducted the random allocation, assigning odd numbers to group A and even numbers to group B. Group A represented the intervention group and group B the control group. To implement this process, 35 random numbers were assigned to each group, and all numbers were placed in sealed envelopes. As each participant arrived, an envelope was drawn at random; if the number was odd, the participant was assigned to the intervention group (group A), and if even, to the control group (group B) (Figure 1).

Despite the researchers’ efforts, participant blinding was not feasible in this study. Only the statistician was unaware of the group assignments, whereas some research staff involved in data collection were not blinded.

The intervention group received five weekly 45 min educational session*s*, while the control group continued receiving routine care (Table 1).

**Table 1:** Brief sessions including dates, time, duration, content, and assessment points.

Steps	Intervention groups	Date	Time (AM)	Duration (min)	Content
First week	First group	Saturday	10:00–10:45	45	Perceived threat
	Second group	Saturday	11:30–12:15	45	Perceived threat
	Third group	Monday	10:00–10:45	45	Perceived threat
	Fourth group	Monday	11:30–12:15	45	Perceived threat
	Fifth group	Wednesday	10:00–10:45	45	Perceived threat
	Sixth group	Wednesday	11:30–12:15	45	Perceived threat

Second week	First group	Saturday	10:00–10:45	45	Perceived self‐efficacy
	Second group	Saturday	11:30–12:15	45	perceived self‐efficacy
	Third group	Monday	10:00–10:45	45	perceived self‐efficacy
	Fourth group	Monday	11:30–12:15	45	Perceived self‐efficacy
	Fifth group	Wednesday	10:00–10:45	45	Perceived self‐efficacy
	Sixth group	Wednesday	11:30–12:15	45	Perceived self‐efficacy

Third week	First group	Saturday	10:00–10:45	45	Guided self‐care action
	Second group	Saturday	11:30–12:15	45	Guided self‐care action
	Third group	Monday	10:00–10:45	45	Guided self‐care action
	Fourth group	Monday	11:30–12:15	45	Guided self‐care action
	Fifth group	Wednesday	10:00–10:45	45	Guided self‐care action
	Sixth group	Wednesday	11:30–12:15	45	Guided self‐care action

Fourth week	First group	Saturday	10:00–10:45	45	Perceived response efficacy
	Second group	Saturday	11:30–12:15	45	Perceived response efficacy
	Third group	Monday	10:00–10:45	45	Perceived response efficacy
	Fourth group	Monday	11:30–12:15	45	Perceived response efficacy
	Fifth group	Wednesday	10:00–10:45	45	Perceived response efficacy
	Sixth group	Wednesday	11:30–12:15	45	Perceived response efficacy

Fifth week	First group	Saturday	10:00–10:45	45	Review and answer to questions
	Second group	Saturday	11:30–12:15	45	Review and answer to questions
	Third group	Monday	10:00–10:45	45	Review and answer to questions
	Fourth group	Monday	11:30–12:15	45	Review and answer to questions
	Fifth group	Wednesday	10:00–10:45	45	Review and answer to questions
	Sixth group	Wednesday	11:30–12:15	45	Review and answer to questions

Ninth week	Control group	Saturday	10:00–12:00	120	Assessment
		Monday	10:00–12:00	120	Assessment
	Intervention group	Thursday	10:00–12:00	120	Assessment
		Wednesday	10:00–12:00	120	Assessment

### 2.1. Setting

The study was conducted at the Deziani Diabetes Clinic in Gorgan City, located in northern Iran.

### 2.2. Measures

Data collection involved three standardized questionnaires:

• *Demographic questionnaire*: This questionnaire collected information on age, gender, marital status, duration of disease, and education level.
• *Treatment compliance questionnaire*: The Adherence to Treatment Questionnaire for Chronic Diseases developed by Seyed‐Fatemi et al. assessed treatment adherence across seven domains: Making Effort for Treatment (MET), Intention to Take the Treatment (ITT), Adaptability (A), Integrating Treatment with Life (ITWL), Stick to the Treatment (ST), Commitment to Treatment (CT), and Indecisiveness for Applying Treatment (IAT). This 40‐item, 6‐point Likert scale questionnaire demonstrated good content validity (CVI = 0.914) and internal consistency (Cronbach’s alpha = 0.921). Test–retest reliability was established at 0.92. Some items are reverse‐scored, with the highest score representing “completely” and the lowest score (0) representing “not at all.” The scoring is oriented positively, so higher total scores or subscale scores indicate greater adherence among respondents [23].
• *Self-care questionnaire*: The Self‐Care Questionnaire for the Elderl*y* developed by Hemmati Maslak Pak and Hashemlou, based on Orem’s general theory, was employed. This 40‐item instrument uses a 4‐point Likert scale (never, rarely, sometimes, often), with reverse scoring applied to specific items. Total scores range from 40 to 160, with higher scores indicating better self‐care behaviors. Content validity was established using the Lawshe table and the Waltz and Basel Content Validity Index, while construct validity was confirmed by factor analysis. The questionnaire demonstrated good internal consistency, with Cronbach’s alpha of 0.864 [24]. Both the treatment compliance questionnaire and the self‐care questionnaire were developed and validated in Iran, ensuring cultural appropriateness for Iranian populations.

### 2.3. Intervention

The intervention, based on the EPPM, comprised four structured steps:

 
*Step 1: Perceived threat*
 The first step *focused* on enhancing perceived threat and included the following activities:
• Discussion of diabetes‐related complication*s* (e.g., kidney damage, eye injuries, cardiovascular disease, and foot ulcers).
• Facilitation of group discussions and responses to questions regarding personal risk.
• Sharing of patient experiences related to diabetes management.
• Encouraging open dialogue about patients’ perceptions of risk.
• Presentation of current diabetes statistics to emphasize prevalence and impact.
 Step 2: *Perceived* self‐efficacy
 Activities in this *step* aimed to enhance perceived self‐efficacy:
• Discussion of potential challenges in diabetes self‐management (e.g., dietary changes, exercise, and habit modification).
• Education on the consequences of uncontrolled diabetes (e.g., nephropathy, retinopathy) and the benefits of effective management.
• Group sharing of successful self‐management strategies and addressing doubts.
• Collaborative problem‐solving to overcome obstacles and barriers to self‐care.
• Building confidence through shared solutions and expert guidance.
 
*Step* 3: Guided self‐care action
 Building on the previously established perceptions of threat and self‐efficacy, participants received education on common diabetes symptoms (e.g., thirst, excessive urination, and hypo‐ and hyperglycemia), self‐management strategie*s*, and problem‐solving techniques. Group problem‐solving sessions were conducted to identify and address individual challenges. Participants shared strategies that enhance self‐efficacy, such as proper nutrition and blood glucose monitoring. Hands‐on skill‐building activities, including insulin injection training, were provided. Self‐efficacy was further reinforced through task decomposition, repeated practice, and ongoing encouragement.
 Step 4: Perceived response efficacy
 The final step focused on enhancing participants’ belief in the effectiveness of the behaviors learned for preventing diabetes complications. Participants were guided to understand the protective role of self‐care and adherence to treatment recommendations in mitigating disease progression. Ongoing process evaluation was conducted through question‐and‐answer sessions, and participant concerns were addressed to reinforce learning throughout the program. Four weeks after the final training session, participants completed a posttest identical to the pretest, with follow‐up appointments scheduled to facilitate this assessment.

### 2.4. Ethical Consideration

The study was conducted according to the guidelines of the Declaration of Helsinki. This study was approved by the Bioethics Committee Research of Golestan University of Medical Sciences in Iran, and its code is IR.GOUMS.REC.1401.046. The study was then registered in the Iranian Registry of Clinical Trials (IRCT ID: IRCT20220522054962N1). Recruitment began at the Deziani Diabetes Clinic on June 9, 2022‐06‐09. The researchers obtained written informed consent from patients for their participation in the study. The privacy and confidentiality of participants were strictly maintained throughout the research. Numerical codes were assigned to participants to ensure data confidentiality, and participants were free to withdraw from the study at any time without consequence.

### 2.5. Data Analysis

Data were analyzed using SPSS version 21. Descriptive statistics (mean, standard deviation, frequency, percentage) were computed for appropriate variables. The Shapiro–Wilk test assessed normality (Table 2). Parametric or nonparametric tests were employed based on data distribution.

**Table 2:** The Shapiro–Wilk test for adherence.

Dimensions	Groups	Statistic	df	*p* value
*Preintervention*				
MET	Intervention	0.964	35	0.307
	Control	0.942	35	0.065

ITT	Intervention	0.969	35	0.404
	Control	0.882	35	0.001

Adaptation	Intervention	0.978	35	0.679
	Control	0.949	35	0.104

ITWL	Intervention	0.937	35	0.044
	Control	0.800	35	0.000

ST	Intervention	0.908	35	0.007
	Control	0.911	35	0.008

CT	Intervention	0.935	35	0.041
	Control	0.872	35	0.001

IAT	Intervention	0.936	35	0.041
	Control	0.856	35	0.000

TOTAL	Intervention	0.841	35	0.000
	Control	0.921	35	0.015

*Postintervention*				
MET	Intervention	0.960	35	0.231
	Control	0.943	35	0.067

ITT	Intervention	0.962	35	0.257
	Control	0.900	35	0.004

Adaptation	Intervention	0.967	35	0.359
	Control	0.896	35	0.003

ITWL	Intervention	0.878	35	0.001
	Control	0.744	35	0.000

ST	Intervention	0.962	35	0.264
	Control	0.949	35	0.104

CT	Intervention	0.929	35	0.026
	Control	0.814	35	0.000

IAT	Intervention	0.910	35	0.007
	Control	0.868	35	0.001

TOTAL	Intervention	0.983	35	0.842
	Control	0.878	35	0.001

Data were analyzed using SPSS version 21. Descriptive statistics, including means, standard deviations, frequencies, and percentages, were calculated for relevant variables. The Shapiro–Wilk test was used to assess the normality of data distribution (Table 2). Depending on the distribution, either parametric or nonparametric statistical tests were employed.

## 3. Results

### 3.1. Baseline Characteristics

There were no significant differences between the intervention and control groups in terms of age (independent *t*‐test, *p* = 0.18), age group (chi‐squared test, *p* = 0.14), gender (chi‐squared test, *p* = 0.51), marital status (chi‐squared test, *p* = 0.50), place of residence (chi‐squared test, *p* = 0.40), or duration of disease (chi‐squared test, *p* = 0.74). Most participants were married (77% in the intervention group and 74% in the control group). Educational level did not differ significantly between groups (Fisher’s exact test, *p* = 0.18) (Table 3).

**Table 3:** Demographic characteristics of the samples in both intervention and control groups.

Variable	Intervention group *N* (%)	Control group *N* (%)	*p* value
Age			
60–70 years	27 (77)	22 (63)	0.14
More than 70 years	8 (23)	13 (37)	
Gender			
female	19 (54)	20 (57) 15 (43)	0.18
male	16 (46)		
Marital status			
married	27 (77)	26 (74)	0.51
Single/widow	8 (23)	9 (26)	
Duration of disease			
under 10 years	6 (17)	6 (17)	0.74
10–15 years	13 (37)	15 (43)	0.58
15 and up	16 (46)	14 (40)	
Mean ± SD	13.94 ± 4.12	13.42 ± 3.61	
Residency			
city	20 (57)	22 (63)	0.43
village	15 (43)	13 (37)	
Education level			
elementary	21 (60)	16 (46)	0.18
Secondary	6 (17)	9 (24)	
High school	8 (23)	7 (20)	
Academic	0 (0)	3 (10)	

### 3.2. Baseline Treatment Adherence

There was no significant difference in treatment adherence between the intervention and control groups at baseline, as assessed by the Mann–Whitney *U* test (*p* > 0.05) (Table 4).

**Table 4:** Comparison of the dimensions of adherence among study groups before and after intervention.

Dimensions groups	Before					After				
	Intervention	Control	CI 0.95	*p* value	Cohen’s d	Intervention	Control	CI 0.95	*p* value	Cohen’s d
	Mean (SD)	Mean (SD)				Mean (SD)	Mean (SD)			
MET	22.91 (3.29)	21.6 (4.53)	(−0.58, 3.21)	0.170^∗^	0.33	26.08 (3.74)	22.4 (4.48)	(1.72, 5.66)	< 0.001^∗^	0.89
ITT	18.14 (3.15)	17.26 (4.58)	(−2, 2)	0.906^∗∗^	0.014	21.14 (4.05)	17.34 (4.41)	(1, 5)	0.001^∗∗^	0.401
Adaptability	14.91 (2.36)	15.05 (2.31)	(−1.26, 0.97)	0.799^∗^	0.061	17.00 (3.85)	15.37 (2.99)	(0.01, 3)	0.034^∗∗^	0.25
ITWL	10.42 (1.71)	10.74 (2.55)	(−1, 1)	0.986^∗∗^	0.002	11.60 (1.78)	11.08 (2.38)	(−0.001, 1)	0.063^∗∗^	0.22
ST	7.81 (2.12)	7.81 (2.12)	(−0.14, 2)	0.242^∗∗^	0.14	9.00 (2.19)	7.65 (2.28)	(0.135, 2.55)	0.029^∗^	0.53
CT	9.74 (2.35)	9.05 (2.89)	(−0.11, 2)	0.098^∗∗^	0.2	10.77 (2.37)	9.14 (2.37)	(1, 3)	0.002^∗∗^	0.36
IAT	6.54 (2.33)	6.02 (2.26)	(−0.13, 1)	0.229^∗∗^	0.14	7.31 (1.87)	5.51 (2.47)	(1, 3)	< 0.001^∗∗^	0.44
Total	90.22 (7.54)	78.85 (7.13)	(−0.12, 6)	0.055^∗∗^	0.23	102.91 (6.67)	88.51 (7.13)	(11, 18)	< 0.001^∗∗^	0.69

^∗^Student’s *t*‐test.

^∗∗^Mann–Whitney *U* test.

### 3.3. Postintervention Treatment Adherence

Following the intervention, Mann–Whitney *U* tests indicated a significant difference in the overall treatment adherence between groups (*p* < 0.01), favoring the intervention group. However, no significant between‐group difference was observed for the “Integrating Treatment with Life” subscale (*p* = 0.06) (Table 4).

### 3.4. Baseline Self‐Care

The Shapiro–Wilk test indicated that self‐care scores were normally distributed. The independent *t*‐test revealed no significant difference in baseline self‐care scores between the intervention and control groups (*p* > 0.05) (Table 5).

**Table 5:** Comparing the dimensions of self‐care in the elderly groups before and after the intervention stage.

Self‐care dimensions	Before intervention						After intervention					
	Intervention Mean (SD)	Control Mean (SD)	CI 0.95	*t*	*p* value	Cohen’s d	Intervention Mean (SD)	Control Mean (SD)	CI 0.95	*t*	*p* value	Cohen’s d
PS‐C^1^	16.65 (3.95)	15.51 (2.99)	(−0.53, 2.8)	1.36	0.83	0.33	21.74 (3.80)	16.05 (3.38)	(3.96, 7.4)	6.6	0.01	1.57
DS‐C^2^	10.54 (2.34)	10.85 (2.25)	(−1.41, 0.78)	−0.57	0.56	0.14	11.31 (2.50)	11.62 (2.23)	(−1.44, 0.81)	−0.50	0.58	0.14
ES‐C^3^	8.88 (2.06)	9.8 (2.56)	(‐0.19, 2.02)	1.64	0.13	0.39	11.57 (2.73)	9.94 (2.38)	(0.41, 2.85)	2.60	0.01	0.64
SS‐C^4^	16.22 (3.67)	16.71 (3.42)	(‐1.21, 2.17)	0.6	0.56	0.14	17.53 (3.21)	16.42 (3.39)	(‐0.43, 2.71)	1.45	0.15	0.35
DIS‐C^5^	20.00 (4.47)	18.71 (5.14)	(‐3.59, 1.01)	−1.11	0.26	0.27	24.08 (5.66)	20.37 (4.66)	(1.27, 6.15)	3.3	0.003	0.73
Total	71.48 (5.56)	72.42 (3.65)	(‐4.99, 3.44)	0.82	0.40	0.09	86.28 (8.4)	74.42 (6.26)	(8.31, 15.41)	6.66	0.01	1.59

^1^Physical self‐care: PS‐C.

^2^Daily self‐care: DS‐C.

^3^Emotional self‐care: ES‐C.

^4^Social self‐care: SS‐C.

^5^During illness self‐care: DIS‐C.

### 3.5. Postintervention Self‐Care

Following the intervention, there were no significant differences between groups in daily and social self‐care (independent *t*‐tests, *p* = 0.58 and *p* = 0.15, respectively). However, significant between‐group differences were observed in other self‐care dimensions as well as overall self‐car*e* (Table 5).

Analysis of covariance (ANCOVA), controlling for pretest scores, demonstrated a significant effect of the intervention on postintervention self‐care (*p* < 0.01, *η* ^2^ = 0.61), indicating that approximately 61% of the posttest variance was attributable to the intervention (Table 6).

**Table 6:** The effect of the Extended Parallel Process Model on the self‐care of type 2 diabetic elderly.

	Sum of squares	df	Mean squares	*F*	*p* value	Eta
Modified model	5183.75	26	287.98	14.14	*p* < 0.01	0.83
Posttest separator	41,894.04	1	41,894.04	20.05	*p* < 0.01	0.96
Group	1669.04	1	1669.04	7.87	*p* < 0.01	0.61
Error	1038.33	51	20.36			
Sum	458,231	70				
Total	6222.07	69				

## 4. Discussion

Promoting and improving self‐care and treatment adherence through structured educational intervention is a key goal in nursing practice. Evidence shows that self‐care and treatment adherence are essential for managing and controlling type 2 diabetes among the elderly [25, 26]. This clinical trial investigated the effect of an EPPM‐based educational intervention self‐care and treatment adherence in elderly patients with type 2 diabetes. The primary aim was to assess the overall effectiveness of the model on these outcomes. At baseline, self‐care levels were suboptimal in both groups, consistent with previous studies reporting reduced self‐care among the elderly [27]. Similarly, Barbosa da Rocha et al.) reported inadequate overall self‐care, particularly low engagement in physical exercise among individuals with type 2 diabetes [25]. Systematic reviews further indicate that the majority of diabetic patients in low‐ and middle‐income countries do not adhere to recommended self‐care behaviors [28].

Following the intervention, there were significant improvements in the overall self‐care and several specific dimensions, including physical self‐care, emotional self‐care, and self‐care during illness. However, no significant differences were observed in daily self‐care or social self‐care. This may be due to the chronic nature of diabetes and the time‐intensive nature of these behaviors. A study in Saudi Arabia highlighted the correlation between perceived social support and diabetes self‐care activities [29]. The lack of significant change in these dimensions in our study may reflect limited emphasis on social and daily care components within the EPPM‐based training, as well as the dynamic and complex nature of social contexts and daily routines among elderly patients. These findings align with previous research demonstrating the effectiveness of the EPPM in improving self‐care behaviors among diabetic patients [30]. Other nursing interventions have reported improvements in diet, exercise, medication adherence, blood glucose monitoring, and foot care [31]. Conversely, some studies indicate low baseline self‐care among type 2 diabetes patients [32]), highlighting the importance of educational interventions for enhancing self‐care and self‐efficacy [33]. Education‐based models such as the EPPM enhance the understanding of disease processes and promote effective self‐care by increasing awareness and motivation [21].

These findings collectively support the efficacy of the parallel process model in improving diabetes management. Consistent with Zarghami et al., it was indicated that the effectively heightened awareness of diabetes complications and their severity thereby increases perceived risk [34].

Cultural context may influence the way elderly individuals perceive threat and efficacy in EPPM‐based interventions. In Iran, cultural norms, family involvement, and social expectations can affect both the perceived severity of diabetes complications and confidence in self‐care behaviors. For instance, family support may enhance perceived efficacy, whereas cultural beliefs about illness or reliance on traditional remedies might moderate perceived threat. These contextual factors should be considered when designing and implementing EPPM‐based interventions to ensure they are culturally sensitive and effective.

In order to enhance self‐care behaviors and treatment adherence, nurses often utilize various educational models. These interventions provide a structured framework for nurses to follow. To create an effective model, multiple studies and ongoing comparisons are necessary. This study showed that the EPPM was successful in improving different aspects of self‐care and treatment adherence. These dimensions are crucial for enhancing the quality of life for individuals with diabetes. For instance, the intervention had a positive impact on the physical and emotional self‐care of diabetic individuals. Research indicates that self‐care in these areas is essential for managing diabetes effectively. By making necessary modifications and adopting healthier habits, individuals with diabetes can lead a high quality and beneficial life.

The intervention also significantly improved overall treatment adherence among elderly diabetics compared to that of the control group. Improvements were observed in attention to treatment, willingness to participate, adaptability, treatment adherence, commitment, and reduced indecisiveness regarding treatment implementation. However, there was no significant difference in integrating treatment into daily life, likely due to the chronic nature of diabetes and the short 4‐week postintervention follow‐up. Despite this, meaningful improvements were achieved in other adherence domains, and the control group showed only minor enhancements.

In the study, the mean total adherence score increased from 90.22 to 102.9 following the intervention. While a change of about 10 points in treatment adherence may not directly translate to the specific amount of improvement per unit of change in the clinic, it can generally be said that a noticeable change will occur in the patient’s adherence behavior.

The observed effect size of 0.61 is considered medium according to Cohen’s benchmarks. Researchers explained that statistical significance depends on various factors such as the size of the effect, the number of participants in the sample, the research design, and the statistical test being employed. [35]. Cohen suggested that a value of *d* = 0.2 should be considered a “small” effect size, while 0.5 represents a “medium” effect size and 0.8 indicates a “large” effect size [36]. Our finding falls between 0.2 and 0.8; therefore, it can be classified a medium effect size.

Baseline demographic characteristics were similar between groups, consistent with previous findings [37]. Barriers to treatment adherence, such as economic concerns, fear of hypoglycemia, and forgetfulness, reported in other studies [38], may differ due to cultural and lifestyle factors.

Various theoretical models have been used to improve self‐care and adherence in diabetes. For example, educational interventions based on the Health Belief Model have shown significant improvements in treatment adherence constructs [39]. Perceived self‐efficacy and social support account for substantial variation in treatment adherence behaviors, with self‐efficacy being the strongest predictor [40]. The EPPM, alongside models such as social cognitive theory and the Health Belief Model, provides a structured framework for promoting adherence and self‐care, although comparisons of model effectiveness require further research.

The EPPM is one of the most widely used models in educational interventions. Other widely used models include social cognitive theory and the health belief model, which includes constructs such as perceived susceptibility, perceived benefits, cues to action, perceived severity, perceived barriers, and self‐efficacy. The superiority and preference of these models as well as other educational models effective on the behavior of diabetic patients for promoting behaviors such as treatment adherence and self‐care are a complex matter that requires extensive studies and scientific, evidence‐based research comparisons that are beyond the scope of this article. However, what can be inferred from the results of this study is that this intervention had significant effects.

### 4.1. Study Limitations

This study has several limitations. First, the single‐site design may limit generalizability to other populations and healthcare settings. Second, the short follow‐up period of 4 weeks precludes assessment of long‐term adherence and self‐care sustainability. Third, reliance on self‐report measures may introduce response bias, although researchers were present during questionnaire completion to minimize errors. Additionally, the study did not measure individual EPPM components quantitatively, which limits the ability to determine the specific contribution of perceived threat, perceived efficacy, and response efficacy to the observed outcomes. Recognizing these limitations highlights areas for future investigation and strengthens the evidence base for diabetes self‐management interventions.

### 4.2. Study Strengths

Despite its limitations, this study has several strengths. The use of a controlled experimental design enhances internal validity compared to observational studies. Validated instruments were employed to assess treatment adherence and self‐care, increasing reliability. The intervention was grounded in a theoretical framework (EPPM), facilitating interpretation of results. Overall, the study contributes to evidence supporting educational interventions for improving diabetes self‐management among elderly populations.

## 5. Conclusion

This study demonstrated the efficacy of an EPPM‐based intervention in improving treatment adherence and self‐care among elderly individuals with type 2 diabetes. By targeting underlying beliefs, enhancing motivation, and providing practical skills, the intervention led to significant improvements in multiple self‐care domains. These findings underscore the potential of the EPPM as a promising approach for diabetes self‐management. Future research should explore long‐term sustainability, applicability across diverse cultural and socioeconomic contexts, and comparative effectiveness against other educational interventions. Since two self‐care dimensions did not show significant improvement, future interventions should emphasize social aspects and daily care behaviors.

### 5.1. Relevance for Clinical Practice

An EPPM‐based intervention can effectively enhance self‐care and treatment adherence among elderly individuals with type 2 diabetes. By increasing motivation and providing practical skills, improvements were observed in multiple self‐care domains. This approach may serve as a promising strategy for diabetes self‐management in clinical settings, with potential to improve patient outcomes and quality of life.

**Nomenclature**
EPPM: Extended Parallel Process Model
MET: Making Effort for Treatment
ITT: Intention to Take the Treatment
A: Adaptability
ITWL: Integrating Treatment with Life
ST: Stick to the treatment
CT: Commitment to treatment
IAT: Applying Treatment
CVI: Content Validity Index
ANCOVA: Analysis of Covariance

## Ethics Statement

The manuscript was derived from the study as a master’s thesis on the elderly nursing in the Nursing Research Center of the Faculty of Nursing and Midwifery at the Golestan University of Medical Sciences.

The study received ethical approval from the Golestan University of Medical Sciences Research Ethics Committee on number IR.GOUMS.REC.1401.046. All participants gave informed consent for their participation.

## Conflicts of Interest

The authors declare no conflicts of interest.

## Author Contributions

Malihe Kabusi: conceptualization, methodology, writing original draft, project administration, and data collection. Gholam Reza Mahmoodi‐Shan: methodology, writing–review and editing, visualization, and supervision. Abdurrhman Charkazi: methodology and writing–review and editing. Mahin Tatari: statistical analysis and data interpretation.

## Funding

The present study was conducted in the Golestan University of Medical Sciences, Nursing Research Center.

## Data Availability

Data are available in reasonable request from the corresponding author via e‐mail.
